# Evaluation design shapes estimates of cross-donor generalization in single-cell classification of cell-type

**DOI:** 10.64898/2026.10.26.761949

**Published:** 2026-10-27

**Authors:** Xingyi Chen, Jiabei Li, Alexis Battle

## Abstract

**Motivation:**

Single-cell cell-type classifiers are often evaluated using random cell-level splits, although cells from the same donor are not independent. We asked how evaluation design affects estimates of cross-donor generalization.

**Results:**

Across PBMC and lung datasets, random cell-level splitting consistently yielded higher macro F1 than donor-held-out evaluation across multiple expression representations. This gap persisted after batch covariate adjustment and donor-level mixed-effects modeling. In contrast, evaluation gaps were minimal in near-ceiling kidney, pancreas, and blood benchmarks and in a randomized-label floor control. These results show that evaluation bias depends on the underlying classification regime and that donor-held-out evaluation is necessary when claims concern generalization to unseen individuals.

**Availability and Implementation:**

Code and analyses are available on GitHub at https://github.com/danielchen05/scRNA-cross-donor-generalization/ and archived at Zenodo at 10.5281/zenodo.23669184. All data are publicly available and cited.

**Contact:**

ajbattle@jhu.edu

## Full Text Availability

The license terms selected by the author(s) for this preprint version do not permit archiving in PMC. The full text is available from the preprint server.

